# Gut Microbiota, Neuroinflammation, and Autonomic Dysfunction in Neurodegenerative Diseases: A Systematic Review of Mechanistic and Translational Evidence

**DOI:** 10.7759/cureus.114312

**Published:** 2026-08-10

**Authors:** Waqas Alauddin, Chetna Srivastava, Poonam Goyal, Mritunjay Shukla, Deepak K Garg, Brishabh R Prajesh, Soumya Singh, Fahad Idrees Shaikh, Sayali Khairnar

**Affiliations:** 1 Physiology, Venkateshwara Institute of Medical Sciences, Amroha, IND; 2 Pathology, Government Medical College and District Hospital, Bundi, Bundi, IND; 3 Radiation Oncology, Narayana Hospital, Jaipur, IND; 4 Physiology, Naraina Medical College and Research Centre, Kanpur, IND; 5 Pathology, Mahatma Gandhi Medical College and Hospital, Jaipur, IND; 6 Dentistry, Institute of Dental Studies and Technologies, Ghaziabad, IND; 7 Medicine, N.K.P. Salve Institute of Medical Sciences and Research Centre and Lata Mangeshkar Hospital, Nagpur, IND; 8 Musculoskeletal Physiotherapy, Dr. N. Y. Tasgaonkar College of Physiotherapy, Karjat, IND

**Keywords:** alzheimer's disease, autonomic nervous system dysfunction, gut-brain axis, gut microbiomes, neurodegenerative diseases, parkinson's disease

## Abstract

Neurodegenerative disorders, including Parkinson's disease, Alzheimer's disease, multiple system atrophy, and amyotrophic lateral sclerosis, are increasingly recognized as complex conditions arising from interactions among the gut microbiota, immune system, and autonomic nervous system. Growing evidence indicates that disruption of the intestinal microbial ecosystem may contribute to neuroinflammatory processes, autonomic impairment, and progressive neurodegeneration through the microbiota-gut-brain axis, although the underlying mechanisms and their translational implications remain incompletely understood. This systematic review was conducted in accordance with the PRISMA 2020 guidelines to comprehensively evaluate the evidence linking gut microbiota dysbiosis with neuroinflammation and autonomic dysfunction across neurodegenerative diseases. A systematic search of PubMed/MEDLINE, Scopus, Web of Science, EMBASE, EBSCOhost, CINAHL, PsycINFO, CENTRAL, Google Scholar, and major grey literature sources yielded 2,769 records. After duplicate removal and eligibility screening, 62 full-text reports underwent detailed assessment, of which 11 studies met the predefined eligibility criteria and were included in the qualitative synthesis. The evidence encompassed human observational, animal experimental, and in vitro approaches, with several studies integrating complementary clinical, microbiome, and experimental methodologies. Most included studies focused on Parkinson’s disease and multiple system atrophy. Across studies, microbial dysbiosis was characterized by reduced abundance of short-chain fatty acid (SCFA)-producing bacteria alongside increased representation of pro-inflammatory microbial taxa. These microbial alterations were associated with disruption of intestinal barrier integrity, activation of inflammatory signaling pathways, microglial activation, elevated pro-inflammatory cytokine production, α-synuclein aggregation, and autonomic manifestations such as gastrointestinal dysmotility and cardiovascular autonomic dysfunction. Experimental studies provided biological support for the microbiota-gut-brain axis, whereas clinical investigations consistently demonstrated associations without establishing causality. Overall, current evidence suggests that gut microbial dysbiosis is closely associated with neuroinflammation and autonomic dysfunction in neurodegenerative diseases and may represent a promising avenue for biomarker discovery and therapeutic intervention. Nevertheless, the available literature is constrained by methodological heterogeneity, limited sample sizes, and the predominance of observational and preclinical studies. Future large-scale longitudinal investigations and rigorously designed randomized controlled trials are required to determine causal relationships and define the clinical utility of microbiome-targeted strategies.

## Introduction and background

Neurodegenerative disorders, including Parkinson's disease (PD), Alzheimer's disease (AD), multiple system atrophy (MSA), and amyotrophic lateral sclerosis (ALS), represent a major cause of disability and death worldwide [1-3]. These disorders are characterized by progressive neuronal loss, accumulation of misfolded proteins, chronic neuroinflammation, and deterioration of motor, cognitive, and autonomic functions. Despite advances in understanding their molecular pathology, the precise mechanisms underlying disease initiation and progression remain incompletely understood, limiting the development of effective disease-modifying therapies [1-3]. Recent evidence has shifted the traditional brain-centered view of neurodegeneration toward a more systemic perspective that recognizes the importance of interactions between the gastrointestinal tract, immune system, and central nervous system [1-3].

The microbiota-gut-brain axis has emerged as a dynamic bidirectional communication network through which intestinal microorganisms and the central nervous system interact via neural, immune, endocrine, and metabolic pathways [4-6]. Gut microorganisms influence brain function by producing bioactive metabolites, regulating intestinal barrier integrity, modulating systemic and central immune responses, and interacting with the autonomic nervous system, particularly through vagal signaling [4-6]. Conversely, the central nervous system can alter gut microbial composition through autonomic regulation of gastrointestinal motility, secretion, and mucosal immunity. Disruption of this complex communication network has been increasingly implicated in the pathogenesis of several neurological and psychiatric disorders [4-6].

Among the proposed mechanisms, gut microbial dysbiosis has emerged as a key contributor to neurodegeneration. Studies have consistently demonstrated that patients with neurodegenerative diseases exhibit reduced microbial diversity and loss of beneficial short-chain fatty acid (SCFA)-producing bacteria, including *Faecalibacterium*, *Roseburia*, and *Blautia*, together with an increased abundance of pro-inflammatory microbial taxa [7,8]. SCFAs, particularly butyrate, play an essential role in maintaining intestinal epithelial integrity, regulating immune homeostasis, and suppressing excessive inflammatory responses [7,8]. Loss of these protective microbial metabolites may increase intestinal permeability, facilitate translocation of microbial products such as lipopolysaccharide, and promote chronic systemic inflammation that ultimately affects the central nervous system [2,7,8].

Neuroinflammation has become increasingly recognized as a central mechanism linking gut microbial alterations to neurodegenerative pathology. The stimulation of microglia and astrocytes, the overproduction of pro-inflammatory cytokines, and the impairment of innate immune communication together lead to neuronal impairment and advancing neurodegeneration [9-12]. Experimental evidence suggests that microbial metabolites and bacterial components activate inflammatory pathways through toll-like receptors (TLRs) and inflammasome signaling, thereby promoting α-synuclein aggregation, neuronal injury, and disease progression. Although these mechanisms have been most extensively studied in PD, similar inflammatory pathways have also been implicated in AD, ALS, and other neurodegenerative disorders [9-12].

In addition to neuroinflammation, autonomic dysfunction represents an important but often underrecognized feature of neurodegenerative diseases. Gastrointestinal dysmotility, constipation, orthostatic hypotension, urinary dysfunction, and cardiovascular autonomic abnormalities frequently occur years before the onset of classic neurological symptoms, particularly in PD and MSA [13-15]. These early manifestations have led to the hypothesis that pathological processes may originate within the enteric nervous system before propagating to the brain via vagal neural pathways. Although this "gut-first" hypothesis remains under investigation, accumulating clinical and experimental evidence supports a close relationship between autonomic dysfunction, intestinal dysbiosis, and progressive neurodegeneration [13-15].

Interest in the microbiota-gut-brain axis has also generated considerable enthusiasm for translational applications. Gut microbial profiles and microbial metabolites have been investigated as potential biomarkers for early diagnosis, disease stratification, and therapeutic monitoring. Likewise, therapies aimed at modulating the gut microbiota, including dietary modification, probiotics, prebiotics, and fecal microbiota transplantation, have shown encouraging results in preclinical studies. However, human evidence remains limited and heterogeneous, and the clinical utility of these approaches has not yet been established [16,17].

Although numerous studies have examined individual aspects of gut microbiota, neuroinflammation, or autonomic dysfunction, these findings have largely been reported in isolation. A comprehensive synthesis integrating mechanistic evidence from experimental studies with observational clinical evidence is needed to better understand their interrelationship and translational relevance across neurodegenerative diseases. Accordingly, this systematic review was undertaken to comprehensively synthesize the existing evidence on the relationship between gut microbiota dysbiosis, neuroinflammation, and autonomic dysfunction in neurodegenerative diseases. Specifically, we synthesized findings from human observational, animal, and in vitro studies to examine the mechanistic pathways linking intestinal dysbiosis with neurodegeneration, evaluate the role of autonomic dysfunction within the microbiota-gut-brain axis, and identify emerging opportunities for microbiome-based diagnostic and therapeutic strategies.

## Review

Methods

This review was performed following the Preferred Reporting Items for Systematic Reviews and Meta-Analyses (PRISMA) 2020 guidelines [18]. The review aimed to systematically evaluate the mechanistic and translational evidence linking gut microbiota alterations, neuroinflammatory processes, and autonomic dysfunction in neurodegenerative diseases. The research question was formulated using the PICO (population, intervention, comparison, and outcome) framework, focusing on the association between gut microbiota dysbiosis and neuroinflammation, their influence on autonomic nervous system regulation, and the resulting impact on disease pathophysiology and progression in neurodegenerative disorders. Particular emphasis was placed on studies investigating microbiota-gut-brain axis mechanisms, neuroimmune interactions, autonomic nervous system alterations, and their translational relevance for diagnosis, prognosis, and therapeutic interventions in neurodegenerative disorders, including PD, AD, MSA, and ALS.

Literature Search Strategy

A comprehensive literature search was conducted to identify studies investigating the relationship between gut microbiota dysbiosis, neuroinflammation, and autonomic dysfunction in neurodegenerative diseases. Electronic databases searched included PubMed/MEDLINE, Scopus, Web of Science, EMBASE, EBSCOhost, CINAHL, PsycINFO, the Cochrane Central Register of Controlled Trials (CENTRAL), and Google Scholar. To minimize publication bias, grey literature sources, including OpenGrey, WorldCat, ClinicalTrials.gov, the World Health Organization International Clinical Trials Registry Platform (WHO ICTRP), PROSPERO, the Open Science Framework (OSF), the ISRCTN Registry, the Chinese Clinical Trial Registry (ChiCTR), the Australian New Zealand Clinical Trials Registry (ANZCTR), and the University Hospital Medical Information Network (UMIN), were also searched [19-21].

The search included studies published between January 2016 and January 2026, without geographical restrictions. Controlled vocabulary terms (MeSH and Emtree) and free-text keywords were combined using Boolean operators (AND and OR). The principal search terms included "gut microbiota," "microbiome," "gut-brain axis," "neuroinflammation," "autonomic dysfunction," "Parkinson's disease," "Alzheimer's disease," "multiple system atrophy," and "amyotrophic lateral sclerosis." Database-specific search strategies were adapted according to the indexing system of each database. The detailed database-specific search strategies are provided in the Appendices.

Eligibility Criteria

Studies were considered eligible if they investigated gut microbiota or microbiome-related alterations in neurodegenerative diseases in relation to neuroinflammatory mechanisms, autonomic dysfunction, or mechanistic and translational pathways relevant to the microbiota-gut-brain axis. Eligible studies included human observational studies, experimental animal studies, and in vitro investigations that provided direct clinical, mechanistic, or translational evidence relevant to the review objectives. Studies were retained when their findings contributed to understanding the interaction between gut microbial alterations and neurodegenerative disease processes, including neuroinflammation, autonomic or gastrointestinal dysfunction, microbial metabolites, gut-brain signaling, or microbiome-mediated therapeutic/drug-related mechanisms. Only original research articles published in English were included. Review articles, systematic reviews, meta-analyses, editorials, conference abstracts, letters, case reports, study protocols, duplicate publications, studies lacking assessment of gut microbiota or microbiome-related outcomes, studies that did not provide relevant neuroinflammatory, autonomic, mechanistic, or translational outcomes, and studies with insufficient data were excluded.

Study Selection

All retrieved records were imported into reference management software, and duplicate records were removed before screening. Two reviewers independently screened titles and abstracts to identify potentially eligible studies. Full-text articles meeting the initial screening criteria were subsequently assessed to determine eligibility according to the predefined inclusion and exclusion criteria. Any disagreements between reviewers were resolved through discussion and, when necessary, consultation with a third reviewer until consensus was achieved.

Data Extraction

Data extraction was independently performed by two reviewers using a standardized data extraction form. The extracted data included the first author, year of publication, country, study design, disease type, study population or experimental model, sample size, gut microbiota characteristics, neuroinflammatory findings, autonomic outcomes, major mechanistic observations, and principal conclusions. Any inconsistencies identified during data extraction were addressed through discussion until consensus was reached.

Quality Assessment and Risk of Bias

Methodological quality was assessed using validated tools appropriate for each study design. Human observational studies were evaluated using the Newcastle-Ottawa Scale (NOS), and the methodological quality of animal studies was evaluated using the Systematic Review Centre for Laboratory Animal Experimentation (SYRCLE) Risk of Bias Tool [22,23]. Two reviewers independently performed the quality assessment, and any differences in judgment were resolved through consensus.

Certainty of Evidence

The certainty of evidence for the primary outcomes was evaluated using the Grading of Recommendations Assessment, Development, and Evaluation (GRADE) approach. The certainty of evidence was categorized as high, moderate, low, or very low based on study design, risk of bias, inconsistency, indirectness, imprecision, and publication bias [24].

Data Synthesis

Owing to substantial heterogeneity in study designs, participant characteristics, experimental models, microbiome profiling techniques, autonomic function assessments, and reported outcomes, a quantitative meta-analysis was not feasible. Therefore, a narrative synthesis was undertaken to integrate and summarize the available evidence. The findings were organized into the principal mechanistic domains identified across the included studies, namely, gut microbiota dysbiosis, neuroinflammation, autonomic dysfunction, microbiota-gut-brain axis mechanisms, and their translational implications, including the potential role of microbiome-based biomarkers and therapeutic interventions in neurodegenerative diseases.

Ethical Considerations

Since this systematic review relied solely on published literature and involved no direct participation of humans or animals, institutional ethics committee approval and informed consent were not required. The review protocol was prospectively registered with the International Prospective Register of Systematic Reviews (PROSPERO) under registration number CRD420261435297.

Results

Study Identification

**Figure 1:**
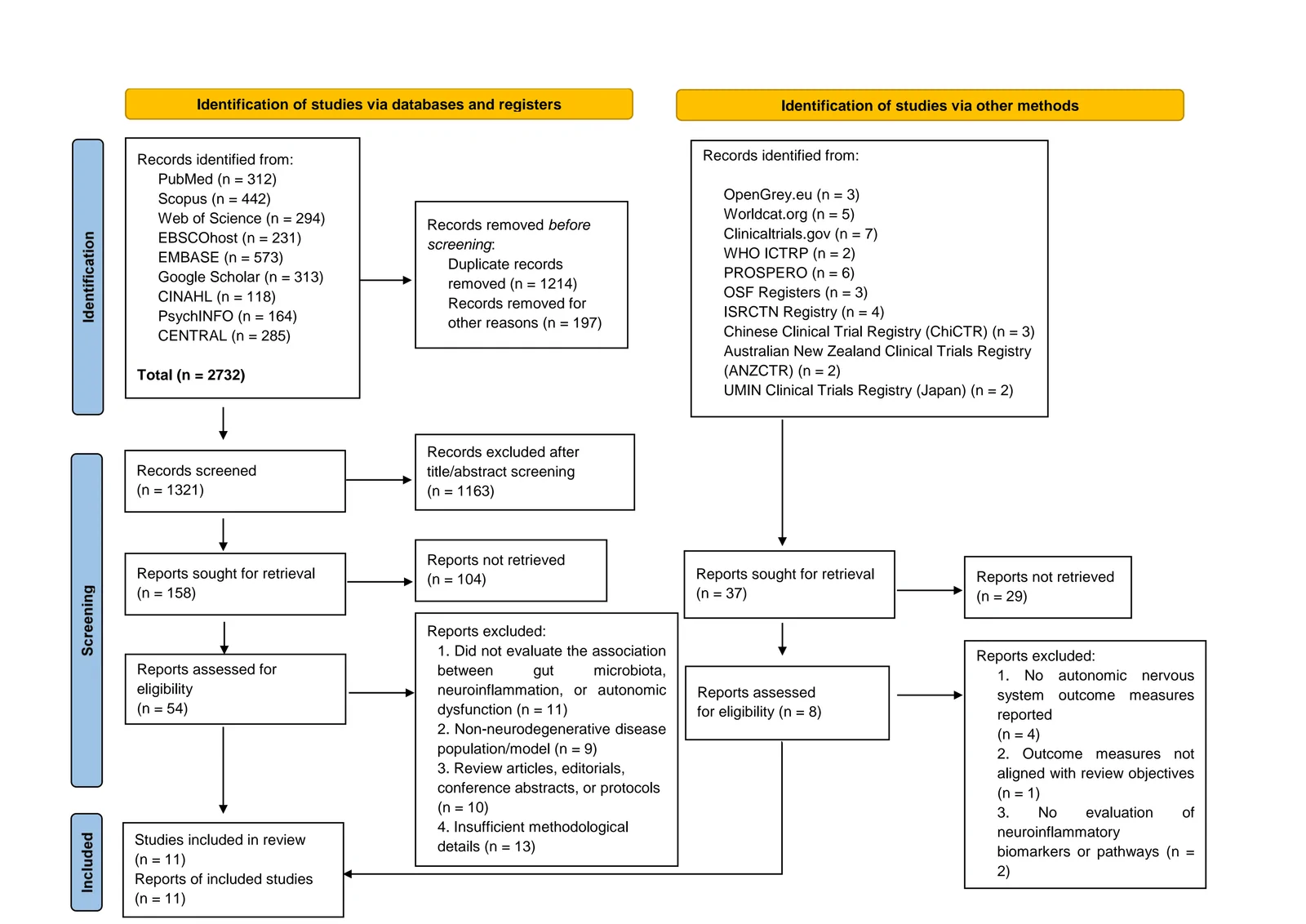
PRISMA 2020 flowchart depicting the study selection workflow. Abbreviations: PRISMA, Preferred Reporting Items for Systematic Reviews and Meta-Analyses; WHO ICTRP, World Health Organization International Clinical Trials Registry Platform; OSF, Open Science Framework; ISRCTN, International Standard Randomised Controlled Trial Number; ChiCTR, Chinese Clinical Trial Registry; ANZCTR, Australian New Zealand Clinical Trials Registry; UMIN, University Hospital Medical Information Network.

The study selection process is summarized in Figure 1. A total of 2,769 records were identified through electronic database searches and other methods, comprising 2,732 records from electronic databases and 37 records from other methods. After removing 1,214 duplicate records and 197 records excluded for other reasons, 1,321 records remained for title and abstract screening. Following screening, 1,163 records were excluded, and 195 reports were sought for retrieval (158 from electronic databases and 37 from other methods). Of these, 133 reports (104 and 29, respectively) could not be retrieved, leaving 62 full-text reports (54 and 8, respectively) for eligibility assessment. Following full-text eligibility assessment, 51 reports were excluded because they did not evaluate the association between gut microbiota, neuroinflammation, and autonomic dysfunction (n = 11), included non-neurodegenerative populations or models (n = 9), represented inappropriate publication types, including review articles, editorials, conference abstracts, or study protocols (n = 10), lacked sufficient methodological information or an accessible full text (n = 13), did not report autonomic nervous system outcome measures (n = 4), reported outcomes not aligned with the review objectives (n = 1), did not evaluate neuroinflammatory biomarkers or pathways (n = 2), or provided insufficient data for extraction (n = 1). Ultimately, 11 studies met the predefined eligibility criteria and were included in the qualitative synthesis (Figure 1) [25-35].

Characteristics of Included Studies

Table 1 summarizes the key characteristics of the 11 included studies. The evidence encompassed human observational, animal experimental, and in vitro approaches, with several studies incorporating complementary clinical, microbiome, and experimental methodologies. Most studies focused on PD and MSA, whereas evidence for other neurodegenerative disorders was limited. The included studies varied in the specific components of the microbiota-gut-brain framework that they assessed. Human observational studies primarily investigated alterations in gut microbial composition in relation to disease-related clinical features, neuroinflammatory biomarkers, gastrointestinal manifestations, and, in several studies, autonomic dysfunction. Animal and in vitro investigations primarily provided mechanistic evidence concerning microbiota-mediated neuroinflammation, α-synuclein pathology, gut-brain signaling, and cellular inflammatory responses. Sample sizes, microbiome profiling techniques, experimental models, and outcome measures varied considerably across studies. Accordingly, individual studies were interpreted according to the outcomes and mechanisms they directly evaluated, rather than assuming that all included studies assessed gut microbiota, neuroinflammation, and autonomic dysfunction simultaneously.

**Table 1:** Summary of included studies. Abbreviations: PD, Parkinson's disease; AD, Alzheimer's disease; MSA, multiple system atrophy; ALS, amyotrophic lateral sclerosis; HC, healthy controls; SCFA, short-chain fatty acids; HRV, heart rate variability; GI, gastrointestinal; FMT, fecal microbiota transplantation; TNF, tumor necrosis factor; BBB, blood-brain barrier; SCOPA-AUT, Scale for Outcomes in Parkinson's Disease for Autonomic Dysfunction; AUC, area under the curve.

Author (year)	Disease	Study design	Sample size	Gut microbiota & neuroinflammatory findings	Autonomic findings	Key mechanistic/Translational evidence
Sampson et al., 2016 [25]	Parkinson's disease	Experimental animal study (FMT)	α-syn mice + fecal microbiota from 6 PD and 6 controls	Gut microbiota promoted α-synuclein pathology, microglial activation, neuroinflammation, and motor deficits; fecal microbiota from PD patients enhanced motor impairment in α-synuclein-overexpressing mice.	Not directly assessed; gastrointestinal dysfunction reported	Demonstrated a causal role of gut microbiota and microbial metabolites in modulating neuroinflammation, α-synuclein pathology, and motor dysfunction in experimental Parkinson's disease
Du et al., 2019 [26]	Multiple system atrophy	Case-control (16S rRNA)	40 MSA + 40 controls (validation cohort included)	Altered fecal and blood microbiota associated with disease	Autonomic failure correlated with SCOPA-AUT scores	Combined microbial signatures differentiated MSA from controls (AUC: 0.853)
Wan et al., 2019 [27]	Multiple system atrophy	Case-control metagenomic study	15 MSA + 15 controls	Gut dysbiosis with altered inflammatory pathways	Severe autonomic dysfunction and constipation	Supports gut microbiota involvement in MSA pathogenesis
Wallen et al., 2020 [28]	Parkinson's disease	Large microbiome-wide association study	855 participants	Depletion of SCFA-producing bacteria and enrichment of opportunistic pathogens	Constipation and GI dysfunction	Supports Braak's gut-origin hypothesis
Wallen et al., 2022 [29]	Parkinson's disease	Shotgun metagenomic case-control study	490 PD + 234 controls	Widespread dysbiosis with immunogenic microbes and inflammatory pathways	Marked GI autonomic dysfunction	Identified multiple microbiome-mediated pathways promoting PD
Cirstea et al., 2023 [30]	Parkinson's disease	Clinical cohort + microbiome/metabolomics + in vitro experimentation	197 PD + 103 controls	Bifidobacterium abundance was associated with levodopa exposure; in vitro experiments demonstrated Bifidobacterium-mediated levodopa metabolism	Not evaluated	Demonstrated microbiome-mediated levodopa metabolism and a functional gut microbiome–drug interaction relevant to Parkinson's disease
Hurley et al., 2023 [31]	Parkinson's disease	In vitro study	STC-1 enteroendocrine cells	Bacterial metabolites activated TLR2/TLR4, increased TNF and α-synuclein	Vagus-mediated gut–brain signaling	Supports gut-first hypothesis through enteroendocrine–vagal communication
Ledonne et al., 2023 [32]	Parkinson's disease	Transgenic animal study	α-syn transgenic rats	Chronic α-syn accumulation caused neurodegeneration	Autonomic involvement discussed	Demonstrated neuronal adaptations during PD progression
Muñoz-Pinto et al., 2024 [33]	Parkinson's disease	Human microbiome profiling + FMT mouse model	44 PD + 21 controls; FMT from 5 PD donors	PD microbiota induced intestinal inflammation, immune activation, α-syn accumulation, and BBB disruption	Enteric and vagal dysfunction	Strong causal evidence linking PD microbiota to neurodegeneration
Park et al., 2024 [34]	Parkinson's disease (body-first vs. brain-first)	Cross-sectional metagenomics	36 PD + 36 controls	Increased Escherichia coli and reduced SCFA-producing bacteria; activated TLR pathways	Greater constipation and autonomic symptoms in body-first PD	Supports gut-origin pathology and microbiome-targeted interventions
Liu et al., 2025 [35]	Multiple system atrophy	Hospital-based case-control	26 MSA, 66 PD, 27 controls	Pro-inflammatory microbiome with altered SCFA pathways and TLR4 activation	High prevalence of constipation (79.2%)	Suggests gut dysbiosis promotes α-synuclein propagation via the vagus nerve

Importantly, the included studies did not uniformly assess all components of the review framework. Some human studies directly evaluated gastrointestinal or autonomic manifestations alongside microbiome alterations, whereas animal and in vitro studies primarily contributed mechanistic evidence concerning neuroinflammation, α-synuclein pathology, gut-brain signaling, and microbiota-mediated cellular responses. Cirstea et al. (2023) provided complementary translational evidence by demonstrating microbiome-mediated levodopa metabolism through clinical microbiome analysis and in vitro experimentation [30]. Accordingly, findings from individual studies were interpreted according to the outcomes actually assessed, and studies without direct autonomic measurements were not considered direct evidence of autonomic dysfunction.

Methodological Quality Assessment

The methodological quality of the included studies was generally high. Human observational studies assessed using the NOS demonstrated a low risk of bias, with five studies achieving the maximum score of 9/9 and one study scoring 8/9. Animal studies assessed using the SYRCLE Risk of Bias Tool showed acceptable methodological quality, although several domains related to randomization, allocation concealment, and blinding were frequently rated as unclear because of incomplete reporting (Table 2).

**Table 2:** Newcastle-Ottawa Scale (NOS) quality assessment of included case-control studies.

Study	Selection (4)	Comparability (2)	Exposure (3)	Total score (/9)	Overall quality
Du et al., 2019 [26]	4	2	3	9/9	High methodological quality with a low risk of bias
Wan et al., 2019 [27]	4	2	3	9/9	High methodological quality with a low risk of bias
Wallen et al., 2020 [28]	4	2	3	9/9	High methodological quality with a low risk of bias
Wallen et al., 2022 [29]	4	2	3	9/9	High methodological quality with a low risk of bias
Park et al., 2024 [34]	4	1	3	8/9	High methodological quality with a low risk of bias
Liu et al., 2025 [35]	4	2	3	9/9	High methodological quality with a low risk of bias

Summary of Mechanistic Findings

A narrative synthesis identified six major mechanistic domains across the included studies (Table 3). Gut microbial dysbiosis was consistently characterized by reduced abundance of SCFA-producing bacteria, particularly *Faecalibacterium*, *Roseburia*, and *Blautia*, together with enrichment of pro-inflammatory taxa such as *Escherichia coli*, *Akkermansia*, and members of the *Enterobacteriaceae*. These alterations were associated with impaired intestinal barrier function, activation of TLR signaling, microglial activation, increased production of pro-inflammatory cytokines, α-synuclein aggregation, gastrointestinal dysmotility, vagal dysfunction, and cardiovascular autonomic abnormalities. Experimental studies provided mechanistic support for the microbiota-gut-brain axis, whereas observational studies demonstrated consistent associations but were insufficient to establish causality. Several studies also highlighted the translational potential of microbiome-derived biomarkers and microbiota-targeted interventions, including dietary modulation, probiotics, and fecal microbiota transplantation (FMT).

**Table 3:** Summary of Key Mechanistic Findings of Included Studies Abbreviations: SCFA, Short-chain fatty acids; BBB, Blood–Brain Barrier; ANS, Autonomic Nervous System; ENS, Enteric Nervous System; TLR, Toll-like receptor; LPS, Lipopolysaccharide; CNS, Central Nervous System; TNF-α, tumor necrosis factor-alpha.

Mechanistic domain	Key findings	Representative studies	Translational significance
Gut microbiota dysbiosis	Consistent depletion of SCFA-producing bacteria (Faecalibacterium, Roseburia, Blautia) and enrichment of pro-inflammatory taxa (Escherichia coli, Akkermansia, Enterobacteriaceae, Lactobacillus).	Sampson et al., 2016 [25]; Wallen et al., 2020 [28]; Wallen et al., 2022 [29]; Muñoz-Pinto et al., 2024 [33]; Park et al., 2024 [34]; Liu et al., 2025 [35]	Suggests dysbiosis is an early contributor to neurodegeneration and a potential diagnostic biomarker.
Neuroinflammation	Dysbiosis was associated with microglial activation, increased TNF-α, IL-1β, IL-6, TLR2/TLR4 signaling, intestinal inflammation, and α-synuclein aggregation.	Sampson et al., 2016 [25]; Hurley et al., 2023 [31]; Ledonne et al., 2023 [32]; Muñoz-Pinto et al., 2024 [33]	Supports chronic neuroimmune activation as a central pathogenic mechanism.
Autonomic dysfunction	Gastrointestinal dysmotility, constipation, vagal dysfunction, and cardiovascular autonomic impairment were consistently reported, particularly in Parkinson's disease and multiple system atrophy.	Du et al., 2019 [26]; Wan et al., 2019 [27]; Wallen et al., 2020 [28]; Park et al., 2024 [34]; Liu et al., 2025 [35]	Indicates autonomic dysfunction may represent an early manifestation of gut-brain axis disruption.
Microbiota-gut-brain axis	Experimental studies demonstrated that gut microbiota regulate neuroinflammation and α-synuclein pathology through vagal signaling, immune activation, and impaired intestinal barrier function.	Sampson et al., 2016 [25]; Hurley et al., 2023 [31]; Muñoz-Pinto et al., 2024 [33]	Provides mechanistic evidence supporting the gut-origin hypothesis of Parkinson's disease.
Microbial metabolites	Reduced SCFA production and altered bacterial metabolites promoted inflammatory signaling, while specific gut bacteria were shown to metabolize levodopa, providing translational evidence of a microbiome-drug interaction.	Wallen et al., 2020 [28]; Wallen et al., 2022 [29]; Cirstea et al., 2023 [30]; Hurley et al., 2023 [31]	Highlights metabolite-based biomarkers and personalized therapeutic approaches.
Therapeutic implications	Probiotics, dietary modulation, fecal microbiota transplantation (FMT), and microbiome-targeted interventions demonstrated potential to modify disease-related pathways in preclinical models.	Sampson et al., 2016 [25]; Cirstea et al., 2023 [30]; Muñoz-Pinto et al., 2024 [33]	Supports further evaluation of microbiome-targeted therapies in clinical trials.

Certainty of Evidence

The certainty of evidence assessed using the GRADE framework ranged from low to moderate (Table 4). Moderate-certainty evidence supported the association between gut microbiota dysbiosis, neuroinflammation, and microbiota-gut-brain axis mechanisms. In contrast, evidence relating to autonomic dysfunction and microbiota-targeted therapeutic interventions was graded as low certainty, primarily because of methodological heterogeneity, reliance on observational studies, small sample sizes, and limited human interventional evidence.

**Table 4:** Certainty of evidence (GRADE summary). Abbreviations: GRADE, Grading of Recommendations Assessment, Development, and Evaluation.

Outcome	No. of studies	Main evidence source	Overall certainty	Reason for downgrading
Gut microbiota dysbiosis in neurodegenerative diseases	11	Human observational + animal studies	Moderate	Heterogeneity among microbiome methodologies
Gut microbiota and neuroinflammation	8	Human and experimental studies	Moderate	Indirectness and limited clinical validation
Gut microbiota and autonomic dysfunction	7	Mainly observational studies	Low	Observational design, inconsistency, small sample sizes
Mechanistic evidence for the microbiota-gut-brain axis	5	Animal and in vitro studies	Moderate	Indirect evidence from experimental models
Microbiota-targeted therapeutic implications	4	Experimental and translational studies	Low	Limited human interventional evidence

Synthesis of Findings

Narrative synthesis identified five major mechanistic themes across the included studies: (1) gut microbiota dysbiosis, (2) neuroinflammation, (3) autonomic dysfunction, (4) microbiota-gut-brain axis mechanisms, and (5) translational implications. Across studies, gut microbial dysbiosis was consistently characterized by depletion of SCFA-producing bacteria and enrichment of pro-inflammatory microbial taxa. These alterations were associated with impaired intestinal barrier integrity, activation of inflammatory signaling pathways, microglial activation, increased production of pro-inflammatory cytokines, α-synuclein aggregation, and autonomic abnormalities, including gastrointestinal dysmotility and cardiovascular autonomic dysfunction. Experimental studies provided mechanistic evidence supporting the microbiota-gut-brain axis, whereas human studies consistently demonstrated associations but were insufficient to establish causality. Emerging translational evidence suggested that microbiome-derived biomarkers and microbiota-targeted interventions may have future diagnostic and therapeutic potential, although further clinical validation is required.

Discussion

This systematic review synthesized the current evidence on the relationship between gut microbiota dysbiosis, neuroinflammation, and autonomic dysfunction in neurodegenerative diseases. Across the 11 included studies, comprising human observational, animal, and in vitro investigations, a consistent association was observed between alterations in gut microbial composition and pathological processes implicated in neurodegeneration. Although most studies focused on PD and MSA, the collective evidence supports the concept that disruption of the microbiota-gut-brain axis contributes to chronic neuroinflammation and autonomic dysfunction, both of which are increasingly recognized as important components of neurodegenerative disease pathogenesis. However, the available human evidence remains predominantly observational, and causal relationships have yet to be established [2,4,5].

One of the principal findings of this review was the consistent identification of gut microbial dysbiosis across the included studies. Human studies demonstrated reduced abundance of beneficial SCFA-producing bacteria, including *Faecalibacterium*, *Roseburia*, and *Blautia*, together with increased abundance of pro-inflammatory taxa such as *Escherichia coli*, *Akkermansia*, and members of the *Enterobacteriaceae* [2,8,25]. These findings are consistent with previous microbiome studies suggesting that loss of SCFA-producing bacteria impairs intestinal barrier integrity and promotes a pro-inflammatory intestinal environment. SCFAs, particularly butyrate, are known to regulate epithelial barrier function, immune homeostasis, and microglial activity, providing a plausible biological mechanism linking gut dysbiosis with neurodegeneration [2,8,25].

Neuroinflammation emerged as the predominant mechanistic pathway connecting gut microbial alterations with neuronal injury. Experimental studies demonstrated activation of microglia, increased production of pro-inflammatory cytokines, including tumor necrosis factor-alpha (TNF-α), interleukin (IL)-1β, and IL-6, activation of TLR signaling pathways, and enhanced α-synuclein aggregation following alterations in gut microbial composition [9-12]. These observations are supported by previous experimental and translational studies demonstrating that microbial metabolites and bacterial products can activate innate immune pathways and sustain chronic neuroinflammation, ultimately contributing to progressive neuronal degeneration [9-12].

Another important finding was the recurring coexistence of gut microbial dysbiosis with gastrointestinal and autonomic abnormalities in several clinical studies. However, the strength and directness of this relationship varied across studies. Some investigations directly assessed autonomic dysfunction, whereas others primarily provided mechanistic or translational evidence relevant to the microbiota-gut-brain axis. Therefore, the available evidence supports an association and biological plausibility but does not establish a uniform or direct causal relationship between gut microbial alterations and autonomic dysfunction across all included studies. Gastrointestinal dysmotility, constipation, vagal dysfunction, and cardiovascular autonomic abnormalities were frequently reported among patients with PD and MSA. These non-motor manifestations often precede the onset of classical motor symptoms, suggesting that autonomic dysfunction may represent an early manifestation of microbiota-gut-brain axis disruption. Nevertheless, the observational nature of the available clinical studies precludes determination of whether autonomic dysfunction is a direct consequence of gut microbial alterations or reflects parallel disease progression [13-15].

The included animal and in vitro studies further strengthened the biological plausibility of the gut-first hypothesis of PD. Germ-free animal models and FMT experiments demonstrated that transplantation of microbiota from patients with PD induced intestinal inflammation, microglial activation, α-synuclein accumulation, and motor impairment in recipient animals [16,17,25]. Similarly, in vitro studies showed that bacterial metabolites activated TLR2/TLR4 signaling pathways and promoted inflammatory responses within enteroendocrine cells, providing mechanistic evidence for communication between the intestinal microbiota and the central nervous system [16,17,25]. Although these findings strongly support the microbiota-gut-brain axis, translation from experimental models to human disease should be interpreted cautiously because animal models cannot fully reproduce the complexity and heterogeneity of human neurodegenerative disorders [16,17,25].

An important translational implication identified in this review is the potential role of the gut microbiome as both a diagnostic biomarker and therapeutic target. Several studies suggested that disease-specific microbial signatures may facilitate early diagnosis or disease stratification [7,32-35]. Furthermore, microbiota-targeted interventions, including probiotics, dietary modification, and FMT, demonstrated beneficial effects in preclinical studies by reducing neuroinflammation and modulating disease-related pathways. Emerging evidence also indicates that gut microorganisms influence levodopa metabolism, highlighting the potential for personalized microbiome-based therapeutic approaches in PD. However, clinical evidence supporting these interventions remains limited, and well-designed randomized controlled trials are required before microbiome-targeted therapies can be recommended for routine clinical practice [7,32-35].

Limitations

This review has several limitations. Only 11 studies met the inclusion criteria, limiting the overall strength of the evidence. Considerable heterogeneity in study design, microbiome profiling methods, autonomic assessments, and reported outcomes precluded quantitative meta-analysis. Moreover, most human evidence was observational, while mechanistic insights were largely derived from animal and in vitro studies, limiting causal inference. Finally, the available evidence was predominantly focused on PD and MSA, with limited data on other neurodegenerative disorders. Despite these limitations, this review integrates findings from human, animal, and in vitro studies, offering an integrated perspective on the interactions among gut microbiota dysbiosis, neuroinflammation, and autonomic dysfunction. Furthermore, the included studies differed in the specific components of the microbiota-gut-brain framework that they assessed. Consequently, individual studies should not be interpreted as demonstrating all three domains of gut microbiota, neuroinflammation, and autonomic dysfunction simultaneously. The findings highlight key mechanistic pathways and identify promising opportunities for microbiome-based diagnostics and therapeutic interventions. Future studies should prioritize large, longitudinal cohorts and well-designed randomized controlled trials using standardized microbiome and autonomic assessment protocols to validate causal relationships and evaluate the clinical utility of microbiome-targeted therapies.

## Conclusions

This systematic review demonstrates that gut microbiota dysbiosis is closely associated with neuroinflammation and autonomic dysfunction across neurodegenerative disorders, particularly PD and MSA. Current evidence indicates that disruption of the microbiota-gut-brain axis may contribute to disease pathogenesis through alterations in microbial composition, immune activation, and autonomic dysregulation. Experimental studies provide important mechanistic insights, whereas current human evidence remains predominantly observational and insufficient to establish causality. Nevertheless, the findings highlight the gut microbiome as a promising source of potential biomarkers and novel therapeutic targets. Collectively, this review underscores the importance of integrating microbiome research into the broader understanding of neurodegenerative diseases and identifies key areas for future investigation. Future large-scale longitudinal studies together with rigorously designed randomized controlled trials are warranted to validate these findings and establish the clinical utility of microbiome-targeted interventions.

## Appendices

**Table 5:** Database-specific search strategies used for the systematic review. Abbreviations: MeSH, Medical Subject Headings; Emtree, Elsevier Life Science Thesaurus; MSA, multiple system atrophy; ALS, amyotrophic lateral sclerosis; WHO ICTRP, World Health Organization International Clinical Trials Registry Platform; OSF, Open Science Framework; ISRCTN, International Standard Randomised Controlled Trial Number; ChiCTR, Chinese Clinical Trial Registry; ANZCTR, Australian New Zealand Clinical Trials Registry; UMIN, University Hospital Medical Information Network; SCI-EXPANDED, Science Citation Index Expanded; SSCI, Social Sciences Citation Index; ESCI, Emerging Sources Citation Index; PROSPERO, International Prospective Register of Systematic Reviews.

Database	Search strategy (Summary)	Filters/Limits
PubMed/MEDLINE	Combined Medical Subject Headings (MeSH) and free-text keywords related to gut microbiota/microbiome, gut-brain axis, neuroinflammation, autonomic dysfunction, and neurodegenerative diseases, including Parkinson's disease, Alzheimer's disease, Multiple System Atrophy (MSA), Amyotrophic Lateral Sclerosis (ALS), Huntington's disease, Lewy body disease, and dementia, using Boolean operators (AND/OR).	English language; human and animal studies; January 1, 2016-January 31, 2026
EMBASE	Combined Emtree terms and free-text keywords related to gut microbiota, microbiome, gut-brain axis, neuroinflammation, autonomic dysfunction, and neurodegenerative diseases, including Parkinson's disease, Alzheimer's disease, Multiple System Atrophy (MSA), Amyotrophic Lateral Sclerosis (ALS), Huntington's disease, Lewy body disease, and dementia, using database-specific syntax and Boolean operators.	Publication years 2016-2026
Scopus	TITLE-ABS-KEY search using keywords related to gut microbiota, microbiome, gut-brain axis, neuroinflammation, autonomic dysfunction, and neurodegenerative diseases, including Parkinson's disease, Alzheimer's disease, Multiple System Atrophy (MSA), Amyotrophic Lateral Sclerosis (ALS), Huntington's disease, Lewy body disease, and dementia.	Publication years 2016-2026
Web of Science	Topic (TS) search combining keywords related to gut microbiota, gut-brain axis, neuroinflammation, autonomic dysfunction, and neurodegenerative diseases, including Parkinson's disease, Alzheimer's disease, Multiple System Atrophy (MSA), Amyotrophic Lateral Sclerosis (ALS), Huntington's disease, Lewy body disease, and dementia, using Boolean operators.	SCI-EXPANDED, SSCI, ESCI; 2016-2026
EBSCOhost (MEDLINE, CINAHL, PsycINFO)	Combined subject headings (MH) and free-text keywords related to gut microbiota, neuroinflammation, autonomic dysfunction, and neurodegenerative diseases, including Parkinson's disease, Alzheimer's disease, Multiple System Atrophy (MSA), Amyotrophic Lateral Sclerosis (ALS), Huntington's disease, Lewy body disease, and dementia.	English language; publication years 2016-2026
CENTRAL (Cochrane Library)	Combined free-text keywords related to gut microbiota, gut-brain axis, neuroinflammation, autonomic dysfunction, and neurodegenerative diseases, including Parkinson's disease, Alzheimer's disease, Multiple System Atrophy (MSA), Amyotrophic Lateral Sclerosis (ALS), Huntington's disease, Lewy body disease, and dementia.	Publication years 2016-2026
Google Scholar	Searches were performed using combinations of keywords including gut microbiota, microbiome, gut-brain axis, neuroinflammation, autonomic dysfunction, Parkinson's disease, Alzheimer's disease, Multiple System Atrophy (MSA), and Amyotrophic Lateral Sclerosis (ALS). The first 200 most relevant records were screened.	January 2016-January 2026
Grey Literature	OpenGrey and WorldCat were searched using combinations of keywords related to gut microbiota, microbiome, gut-brain axis, neuroinflammation, autonomic dysfunction, and neurodegenerative diseases, including Parkinson's disease, Alzheimer's disease, Multiple System Atrophy (MSA), and Amyotrophic Lateral Sclerosis (ALS).	January 2016-January 2026
Clinical Trial Registries	ClinicalTrials.gov, WHO ICTRP, ISRCTN Registry, ChiCTR, ANZCTR, and UMIN were searched using combinations of gut microbiota, microbiome, dysbiosis, Parkinson's disease, Alzheimer's disease, Multiple System Atrophy (MSA), Amyotrophic Lateral Sclerosis (ALS), and related neurodegenerative disease terms.	January 2016-January 2026
Review Registries	PROSPERO and the Open Science Framework (OSF) were searched to identify ongoing or completed systematic reviews investigating gut microbiota, neuroinflammation, autonomic dysfunction, and neurodegenerative diseases, including Parkinson's disease, Alzheimer's disease, Multiple System Atrophy (MSA), and Amyotrophic Lateral Sclerosis (ALS).	January 2016-January 2026
